# Choroidal juxtapapillary neovascularization regression in multiple evanescent white dot syndrome by optical coherence tomography angiography: a case report

**DOI:** 10.1186/s13256-019-2211-8

**Published:** 2019-08-31

**Authors:** Maria Cristina Savastano, Marco Rispoli, Bruno Lumbroso

**Affiliations:** Centro Italiano Macula, Via Angelo Brofferio, 7, 00196 Rome, Italy

**Keywords:** Choroidal neovascularization, Multiple evanescent white dot syndrome, OCT angiography

## Abstract

**Background:**

Multiple evanescent white dot syndrome most often resolves spontaneously without complications; however, choroidal neovascularization can sometimes occur.

**Case presentation:**

Here, we describe a case of a 22-year-old white Caucasian man with blurred vision in his left eye who exhibited juxtapapillary choroidal neovascularization on optical coherence tomography angiography. Although multiple evanescent white dot syndrome is often self-limiting, to reduce the possibility of an inflammatory reaction, we preferred to administer prednisolone orally. After 3 months, significant regression of juxtapapillary neovascularization was observed by B-scan and optical coherence tomography angiography. Symptoms resolved in 3 months. A steady situation was observed at 4 years of follow-up.

**Conclusion:**

This case report highlights the helpful use of optical coherence tomography angiography in daily clinical practice, even in inflammatory diseases, such as atypical juxtapapillary neovascularization in multiple evanescent white dot syndrome. Choroidal neovascularization associated with multiple evanescent white dot syndrome by means of optical coherence tomography angiography showed neovascular activity regression, thus avoiding invasive therapy.

## Background

Multiple evanescent white dot syndrome (MEWDS) is an acute chorioretinal inflammatory disorder that is frequently self-limiting. Usually, MEWDS is a unilateral disease that typically affects young healthy adults and is characterized by the development of gray or white flat lesions in the ellipsoid zone (EZ) at the posterior pole and at the mid-peripheral fundus [1]. The disease onset is variable and usually starts with diminished vision and enlargement of the blind spot. Although the etiology is still unknown, a possible correlation with viral infection has been speculated, as antecedent viral illness has been reported in most cases [2]. The typical placoid lesions appear during the first weeks and resolve spontaneously after a variable period, leading to the typical clinical aspect characterized by mottling, atrophy, and hyperpigmentation of the retinal pigment epithelium (RPE), which is generally associated with good functional recovery. Usually, MEWDS has a good long-term prognosis for visual acuity, although some patients have residual persistent symptoms such as scotomas. In rare cases, MEWDS can generate a severe loss of vision due to irreversible damage to the EZ/RPE, macular atrophic development, or choroidal neovascularization (CNV) development [3]. CNV is an uncommon complication of MEWDS and generally occurs in atypical cases [4]. Fluorescein angiography (FA) is still considered the standard tool for neovessel visualization. Recently, a new dye-free method for retinal vessel analysis has been introduced: optical coherence tomography angiography (OCTA). Although a different method may provide blood flow movement, one studied algorithm is based on split-spectrum amplitude-decorrelation angiography (SSADA) described by Jia *et al.* [5]. SSADA has been successfully reported for retinal circulation assessment in healthy eyes [6] and in different macular diseases, such as CNV [7] and retinal vein occlusion [8]. OCTA allows the detection of blood flow and the observation of the morphology of retinal vessels. The aim of the present report is to describe a rare case of neovascularization regression in an eye with MEWDS assessed by OCTA.

## Case presentation

**Fig. 1:**
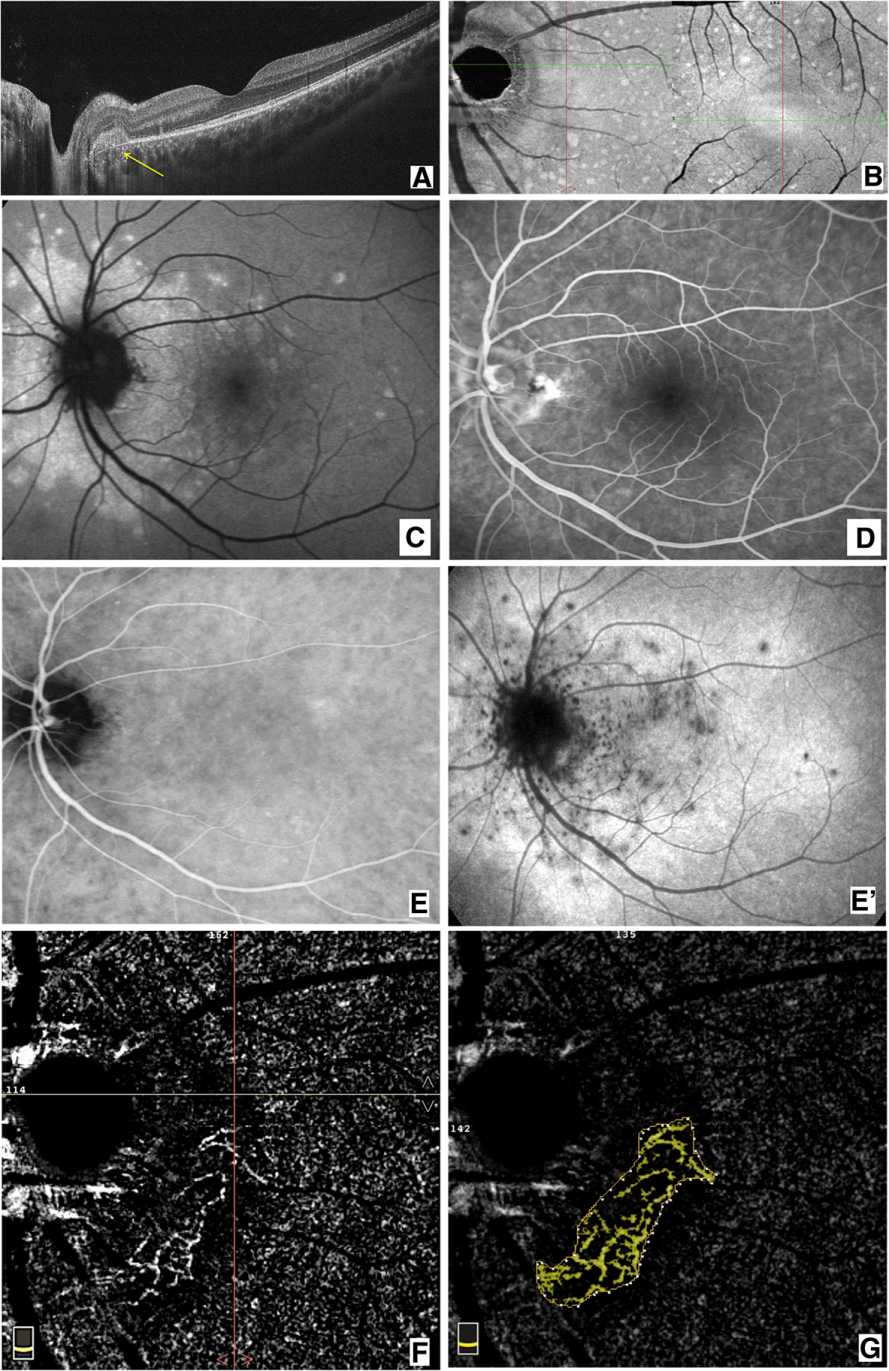
Multiple evanescent white dot syndrome unilateral eye. **a** Optical coherence tomography angiography B-scan shows the hyporeflective area temporally to the optic disc (*yellow arrow*). **b** En face scan corresponding to the retinal pigment epithelium shows diffuse hyperreflective spots. **c** Autofluorescence reveals diffuse hyperautofluorescent areas around the optic disc that extended into the macula region. **d** Fluorescein angiography shows juxtapapillary hyperfluorescent dots of leakage. **e-e’** Indocyanine green angiography similarly shows the hypercyanescent dots temporally in the disc, and in the early and late phase, hypocyanescent dots diffuse at posterior pole with a hyperreflective halo. **f** Optical coherence tomography angiography showed anomalous neovascularization as an arcuate flow vascular net on the temporal side of the disc. Vascular net was formed by thin tangled capillaries. **g** The neovascular area outline for the assessment (*yellow area*)

**Fig. 2:**
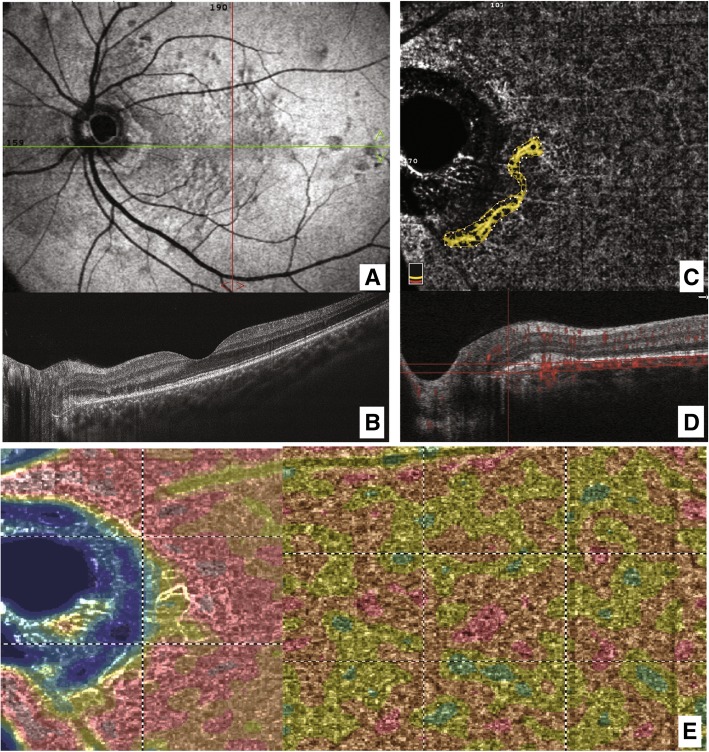
Multiple evanescent white dot syndrome unilateral eye. **a** En face scan corresponding to the retinal pigment epithelium shows a reduction in hyperreflective spots compared to the baseline. **b** The B-scan through the juxtapapillary neovascularization shows regression of hyperreflective spots above the retinal pigment epithelium. **c** Optical coherence tomography angiography neovascular area outline for the assessment (*yellow area*) shows regression compared with the baseline. **d** Reference plane segmentation of the optical coherence tomography angiography image with the flow details (*red dots*). **e** Optical coherence tomography angiography density map of choroidal flow shows dots of flow reduction in the temporal juxtapapillary area. Similarly, microdots of the decreased choroidal flow area are observed at the posterior pole, corresponding to hyporeflectivity of the retinal pigment epithelium in the en face scan

**Fig. 3:**
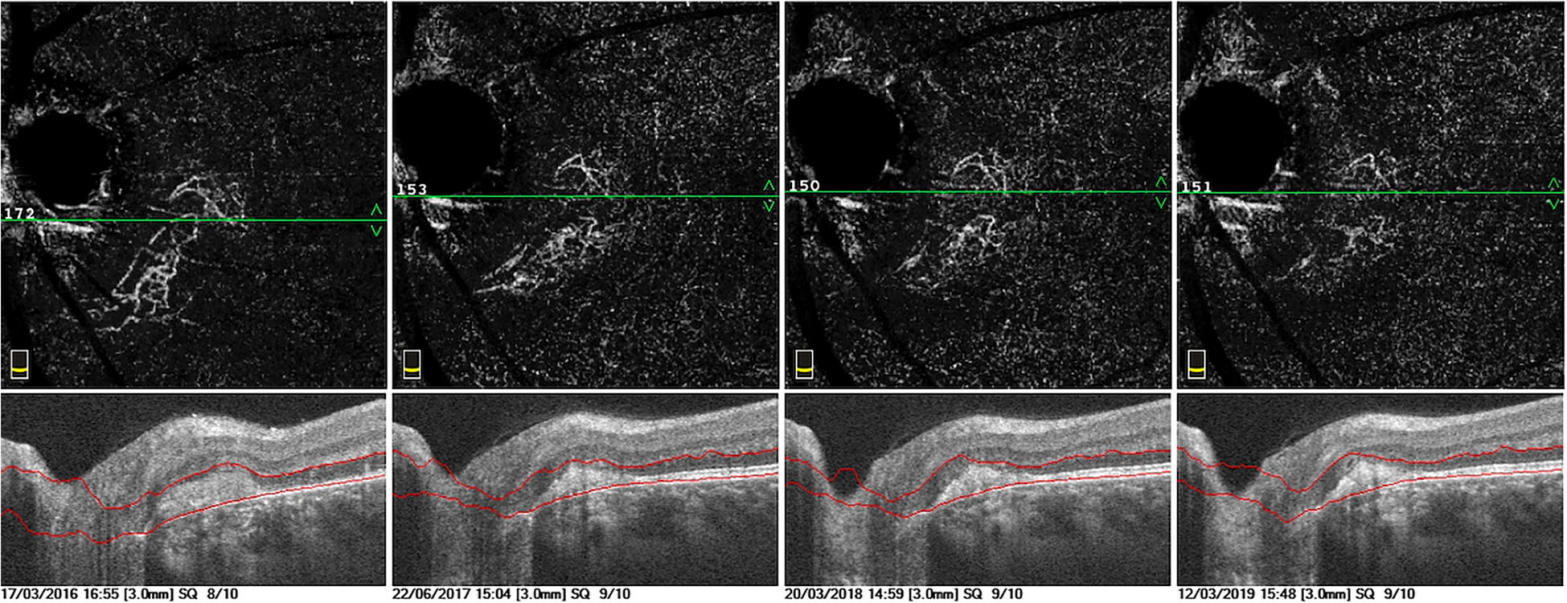
Multiscan view of four different evaluations during 4 years of follow-up. B-scan shows the hyperreflective subretinal materials in the juxtapapillary region that have an irregular profile at baseline that decrease and develop defined borders. In parallel, the corresponding optical coherence tomography angiography shows a reduction in flow in choroidal neovascularization and reorganization of the vessel architecture silhouette over time

A 22-year-old white Caucasian man came to our office complaining of blurred vision in his left eye (LE) with night blindness and difficulty in driving. A flu-like illness was reported 2 weeks before the ocular symptoms began. His best-corrected visual acuity (BCVA) was 20/20 in his right eye (RE) and 20/20 in his LE. A fundus examination showed the healthy condition of his RE, while his LE showed only a scattered mottling of the fundus pigment on the temporal side of the optic nerve. We performed B-scan optical coherence tomography (OCT) around the optic nerve head that showed hyperreflectivity in the outer retina with interruption of the ellipsoid layers in the juxtapapillary region (Fig. 1A). An en face scan showed diffuse hyperreflective spots in the RPE slab (Fig. 1B). After performing autofluorescence (FAF), FA, indocyanine green angiography (ICG), and OCTA, MEWDS was diagnosed (Fig. 1). FAF showed diffuse hyperautofluorescent areas around the optic disc that extended into the macula region (Fig. 1C). The en face scan showed small hyperreflective dots at the posterior pole. The FA (Fig. 1D) revealed juxtapapillary hyperfluorescent dots of leakage similar to the ICG early and late phase (Fig. 1E, E’). Furthermore, the late frames of ICG showed diffuse dots of hypocyanescence at the posterior pole. OCTA showed anomalous neovascularization as an arcuate flow vascular net on the disc temporal side. The vascular net was formed by thin tangled capillaries (Fig. 1F). The neovascular tangled net area was outlined in the assessment (Fig. 1G). Although it is known that MEWDS is often self-limiting, to reduce the possible inflammatory reaction, we preferred to administer prednisolone orally with an initial prescription of 50 mg/day for 7 days, followed by 25 mg/day for 7 days, 12.5 mg/day for 7 days, and 5 mg/day for the last 7 days. After 1 month of therapy, significant regression of juxtapapillary neovascularization was observed. Small capillaries regressed almost completely, leaving only wide and straight main vessels (Fig. 2). Our patient’s symptoms resolved. Figure 3 shows the evolution over a 4-year observation period. A steady situation was observed after 4 years with an absence of symptoms, and BCVA was 20/20.

## Discussion

Inflammatory maculopathies comprise a heterogeneous group of inflammatory syndromes involving the choroid, EZ, RPE, and inner and outer retina. Neovascularization is a rare complication of MEWDS. Juxtapapillary CNVs are even rarer. Multimodal imaging is presently the best way to diagnose and follow the evolution of this disorder. Recently, OCTA has gained great importance in analyzing vascular anomalies, even in MEWDS [9]. According to Nozaki *et al.* [10], the dye leakage and pooling of FA made it impossible to diagnose CNV. However, OCTA can highlight the vascular structure in the outer retina, thereby leading to CNV diagnosis (Fig. 2E). Our study is the first investigation of juxtapapillary neovascularization specifically quantified by OCTA in a rare inflammatory maculopathy, MEWDS. The OCTA allowed us to monitor the CNV activity.

## Conclusions

MEWDS is an inflammatory retinal disorder that involves the outer retina. FA and ICG can be as helpful in the diagnosis as fundus FAF, as described by Furino *et al.* [11].

Recently, the introduction of OCTA has allowed retinal vasculature architecture assessment without dye injection. Our study showed that OCTA imaging analysis can detect neovascularization regression during monitoring in a patient with MEWDS. As previously described by Chen *et al*. [4], CNV can occur in atypical MEWDS at the posterior pole. One of their cases had previously received a single anti-vascular endothelial growth factor (VEGF) injection and showed substantial improvement in visual acuity and anatomic findings after combination therapy with orally administered prednisone. In our case, the CNV was localized to the juxtapapillary region. We observed complete regression of juxtapapillary neovascularization in a young man with atypical MEWDS, suggesting the possibility of neovascular regression in inflammatory eye diseases, thus avoiding invasive therapy.

## Data Availability

Yes, in case of necessity, all data are available for the journal.
